# Application value of multiparametric MRI for evaluating iron deposition in the substantia nigra in Parkinson's disease

**DOI:** 10.3389/fneur.2022.1096966

**Published:** 2023-01-04

**Authors:** Qing Cao, Jinjin Huang, Dongping Tang, Hao Qian, Kun Yan, Xun Shi, Yaowei Li, Jiangong Zhang

**Affiliations:** ^1^Department of Radiology, Guangzhou Xinhai Hospital, Guangzhou, Guangdong, China; ^2^Department of Neurosurgery, The PLA 74th Group Army Hospital of Chinese, Guangzhou, Guangdong, China; ^3^Department of Science and Education Department, Guangzhou Xinhai Hospital, Guangzhou, Guangdong, China; ^4^Department of Neurology, Guangzhou Xinhai Hospital, Guangzhou, Guangdong, China; ^5^Department of Nuclear Medicine, The First People's Hospital of Yancheng, The Fourth Affiliated Hospital of Nantong University, Yancheng, Jiangsu, China

**Keywords:** Parkinson's disease, magnetic sensitivity weighted imaging, T2^*^mapping, image fusion, MR

## Abstract

**Objective:**

This study aimed to investigate the application value of multi-parametric magnetic resonance imaging (MRI) in the diagnosis of iron deposition in the substantia nigra dense zone in Parkinson's disease (PD) and to evaluate the diagnostic value of the correlation among multi-parametric imaging indicators, clinical stage, and disease duration.

**Materials and methods:**

Thirty-six patients with clinically confirmed PD and 36 healthy controls were enrolled. The disease course was recorded, and PD severity was graded using the Hoehn–Yahr (H–Y) scale. All subjects underwent magnetic sensitivity weighted imaging (SWI), neuromelanin magnetic resonance imaging (NM-MRI), and a T2^*^mapping sequence. Based on the fusion of the NM-MRI and SWI amplitude maps, phase maps, and T2^*^MAPPING value maps, NM-MRI was used to delineate the dense zone of the substantia nigra, which was divided into three sub-regions: upper, middle, and lower. In this way, the amplitude, phase, and R2^*^ values of each sub-region and the average value of the sum of the three sub-regions were obtained simultaneously in the SWI amplitude, phase, and T2^*^MAPPING maps. The multi-parameter imaging indices were compared between the two groups, and the correlation between them and clinical indices was evaluated in the PD group.

**Results:**

The upper (amplitude, phase value, R2^*^ value), middle, and lower (amplitude) right substantia nigra compact zones were significantly different between the PD and control groups. The upper (phase value, R2^*^ value) and middle (amplitude) areas of the left substantia nigra compact zone were also significantly different between the two groups (all *P* < 0.05). The mean values (amplitude, phase value, R2^*^ value) of the right substantia nigra densification zone and the mean values (phase value) of the left substantia nigra densification zone were also significantly different (all *P* < 0.05). Amplitudes in the middle and lower parts of the right substantia nigra dense zone were negatively correlated with the H–Y grade (middle: *r* = −0.475, *P* = 0.003; lower: *r* = −0.331, *P* = 0.049). Amplitudes of the middle and lower parts of the dense zone of the left substantia nigra were negatively correlated with the H–Y grade (middle: *r* = −0.342, *P* = 0.041; lower: *r* = −0.399, *P* = 0.016). The average amplitude of the right substantia nigra compact zone was negatively correlated with the H–Y grade (*r* = −0.367, *P* = 0.027). The average R2^*^ value of the compact zone of the left substantia nigra was positively correlated with the H–Y grade (*r* = 0.345, *P* = 0.040).

**Conclusion:**

Multiparametric MRI sequence examination has application value in the evaluation of iron deposition in the dense zone of the substantia nigra in PD. Combined with NM-MRI, fusion analysis is beneficial for accurately locating the substantia nigra compact zone and quantitatively analyzing the iron deposition in different sub-regions. Quantitative iron deposition in the middle and lower parts of the substantia nigra dense zone may become an imaging biological indicator for early diagnosis, severity evaluation, and follow-up evaluation of PD and is thus conducive for clinical diagnosis and treatment evaluation.

## 1. Introduction

Parkinson's disease (PD) is a common condition associated with neurological degeneration, and clinical symptoms are aggravated with an increase in neuronal degeneration (1). Imaging modalities, especially using magnetic resonance imaging (MRI), and new sequences have been used to explore more sensitive imaging biological and clinical indicators for correlation analysis. The main neuropathological changes in patients with PD are damage to the substantia nigra neurons and the related dopamine receptor pathway; an increase in abnormal iron deposition in the substantia nigra area; and an increase in the content of neuromelanin iron complex, which induces glial cell aggregation, thus leading to neuronal damage and accelerated cell death (2, 3). Previous studies have indicated that iron deposition in the substantia nigra is normally concentrated in the reticular zone, while neuromelanin is mainly concentrated in the dense zone of the substantia nigra. In PD patients, the neuromelanin content in the dense zone of the substantia nigra decreases and iron deposition increases, reflecting the degeneration process of dopamine neurons (4). To better understand the association between iron deposition in the substantia nigra compacta and the degeneration of dopamine neurons (5), it is necessary to accurately locate iron deposition in the substantia nigra compacta and evaluate the changes in iron deposition in the substantia nigra compacta (6).

Thus, this study aimed to analyze the differences in iron deposition in the substantia nigra compact zones and the correlation between iron deposition and PD onset, to find more sensitive biological imaging indicators, and to provide new approaches for the imaging diagnosis of PD. This study was based on magnetic sensitivity-weighted imaging (SWI), which has highly sensitive for detecting iron deposition area and its scope (7). Neuromelanin magnetic resonance imaging (NM-MRI) can better show the distribution of neuromelanin in the substantia nigra (8, 9), and T2^*^mapping can be used to quantify iron deposition (10). In this study, these three sequences were fused in pairs, and the substantia nigra was divided into the upper, middle, and lower regions for analysis and comparison.

## 2. Materials and methods

### 2.1. Study design and participants

This prospective study enrolled 36 patients with PD from the Department of Neurology of Guangzhou Xinhai Hospital between March 2020 and August 2022. The inclusion criteria were as follows: (1) PD diagnosis according to the Chinese diagnostic standard (2020 version), that is, the patient has motion retardation and at least static tremor or myotonia; (2) ability to cooperate with MRI examination and no contraindications to MRI scanning; and (3) the image quality meets the evaluation criteria. The exclusion criteria were as follows: (1) secondary Parkinson-like syndrome caused by other organic diseases of the nervous system or drugs and (2) other neurological and psychiatric diseases, trauma, and developmental malformations affecting the image evaluator. The clinical data, medical history, and Hoehn–Yahr (H–Y) grade of the PD patients were collected, and multiparameter MRI images were obtained.

In addition, 36 healthy control subjects were recruited from Guangzhou Xinhai Hospital staff and society. The inclusion criteria were as follows: (1) no PD-related exercise or non-exercise symptoms; (2) no family history of PD and idiopathic tremor; (3) ability to cooperate with magnetic resonance examination and no contraindication to magnetic resonance scanning; and (4) the image quality met the evaluation criteria. The exclusion criteria were: (1) other neurological and psychiatric diseases and taking central medicine; (2) trauma, developmental malformation, and other conditions affecting image evaluation.

This study was approved by the ethics committee of Guangzhou Xinhai Hospital (Approval No.: GZXH-20200147) and was conducted according to the tenets of the Declaration of Helsinki. All subjects and their family members were informed of the purpose of the study, the duration of the scan, no radiation risk, and precautions for examination in detail. Informed consent was obtained from all participants. The general brain conditions were interpreted by him and his family afterwards.

### 2.2. Instrument and imaging

A 3.0T MRI system (United-Imaging Medical, uMR780) and a 24-channel coil for the head and neck were used. Conventional MRI sequence acquisition included transverse axial [T1 weighted imaging (T1WI), T2 weighted imaging (T2WI), fluid attenuated inversion recovery (FLAIR), diffusion-weighted imaging], sagittal (T2WI), coronal (FLAIR), SWI, NM-MRI, and T2^*^mapping sequences. All parameters are listed in Table 1. The total scanning time of the MRI was 28 min 36 s.

**Table 1:** Scan each sequence parameter value.

**Sequence**	**TR (ms)**	**TE (ms)**	**Voxel size (mm)**	**Number of layers**	**Layer spacing (mm)**	**Roll back angle**	**Average times**	**Interpolation**	**IR (ms)**
T2WI_TRA	5,103	119.9	0.67*0.60*5.00	23	1	150	2	–	–
T1WI_TRA	2,267	10.2	0.69*0.63*5.00	23	1	135	1	–	920
FLAIR_TRA	8,000	118.6	0.95*0.76*5.00	23	1	120	2	–	2,500
DWI_TRA	2,087	107.1	1.44*1.44*5.00	23	1	–	–	–	–
T2WI_SAG	5,683	121.8	0.77*0.65*5.00	23	1	145	1.2	–	–
FLAIR_COR	8,000	112.9	0.90*0.72*5.00	25	1	150	1.5	–	2,500
SWI_TRA	30.3	20	0.51*0.51*2.00	56	–	–	1	2	–
NM-MRI_TRA	738	10.54	0.62*0.44*3.00	22	0	130	5	–	–
T2*MAPPING_TRA	436.7	2.24/4.48/6.72/8.96/11.2	0.48*0.48*3.00	11	0.3	–	1	–	–

### 2.3. Image preprocessing analysis

**Figure 1:**
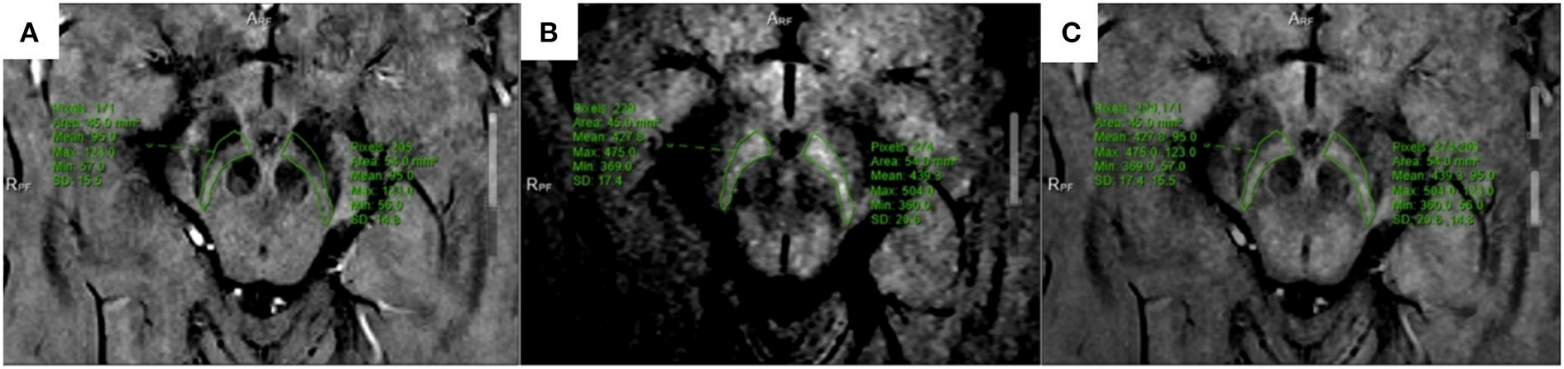
Located in the upper part of SNc. **(A)** SWI-amplitude diagram; **(B)** NM-MRI map; **(C)** The two images are fused, and the corresponding region amplitude is obtained.

**Figure 2:**
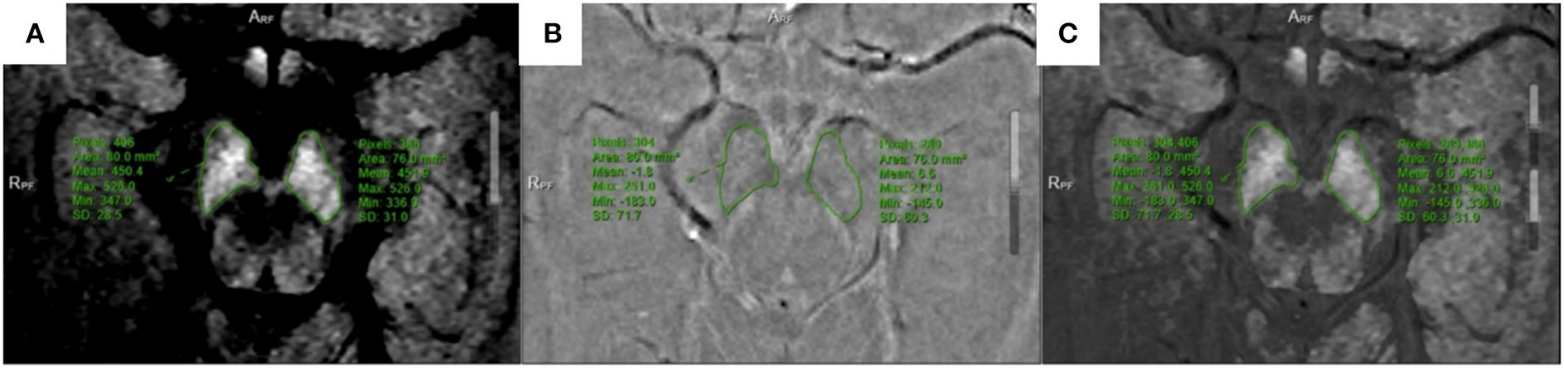
Located in the middle of SNc. **(A)** NM-MRI map; **(B)** SWI-phase diagram; **(C)** The two images are fused, and the phase value is obtained.

**Figure 3:**
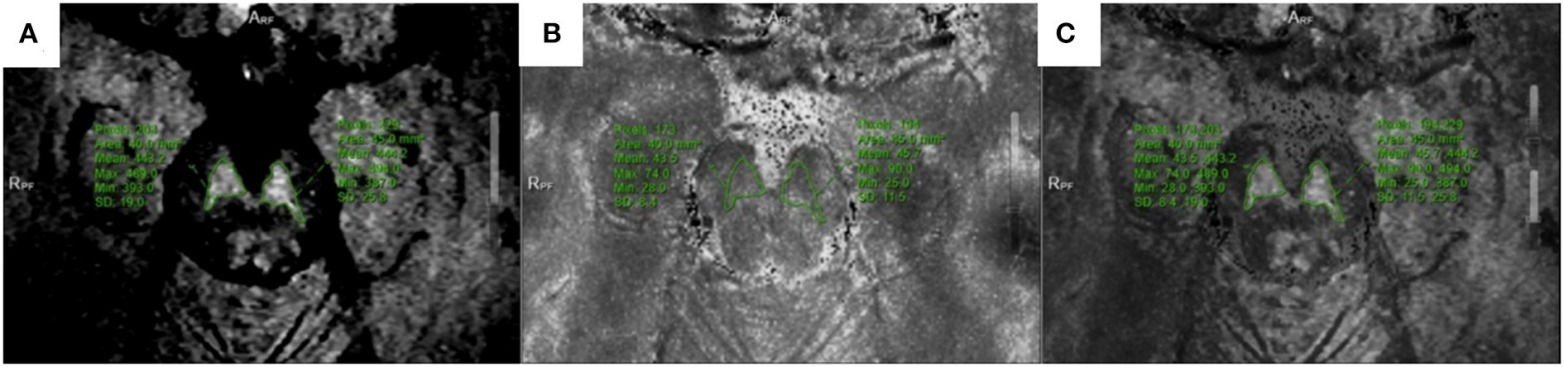
Located in the lower of SNc. **(A)** NM-MRI map; **(B)** T2*mapping; **(C)** Combine the two maps and obtain T2* value.

All multiparameter image data were processed in a 3.0 T post-processing workstation (model: uWS-MR-R004). Two diagnostic imaging physicians with 12 and 20 years of experience independently evaluated the images in a blinded manner before meeting for consensus. They measured the relevant image indicators and considered the average value of the two. NM-MRI was fused with the SWI amplitude, phase, and T2^*^MAPPING maps. Using NM-MRI, the melanin distribution of neurons in the bilateral substantia nigra was delineated layer-by-layer, and the upper, middle, and lower sub-regions were equally divided. The upper layer included the red nucleus and the substantia nigra reticulata with a maximum low signal (Figure 1A), and the middle layer included the area of the substantia nigra compact zone with a high signal. This layer indicated the boundary between the red nucleus and the substantia nigra reticulata and the substantia nigra compact zone and was delineated based on the high NM-MRI signal (Figure 1B). The central layer was located at the largest level of the substantia nigra compact zone based on the high NM-MRI signal at the time of delineation and indicated the boundary between the red nucleus and the substantia nigra reticularis and the substantia nigra compact zone (Figure 2A). The lower layer was located at the caudal level of the substantia nigra, which was also delineated with a NM-MRI hyperintensity (Figure 3A).

The SWI amplitude (Figure 1A), phase value (Figure 2B), and T2^*^ value of the T2^*^maps (R2^*^ value = 1/T2^*^ value, Figure 3B) of the delineated area at each level were converted to radians using the following formula: *f* (*x*) = x^*^π/1,800, where *x* is the actual measured value, and the phase value range is between –π and+π. The measurement and data processing methods were performed under the guidance of the Joint Photographic Engineer. All images were automatically fused using the image fusion function of the post-processing workstation (Figures 1C, 2C, 3C), and the image fusion effect, including the edges of anatomical structures, blood vessels, and 3D MPR reconstruction layers, was evaluated by the two physicians to ensure that the image fusion met the diagnostic requirements. Before sketching, the window width and position were adjusted to be consistent to reduce the difference between the vision and signal.

The boundary of the melanin high-signal area was clear and easily sketched, while the attenuation area was not sketched (11). The region of interest corresponding to the anatomical region of SWI and T2^*^mapping was used to measure its value. The two physicians easily reached a consensus on the boundary of the region of interest with high operability and repeatability. Images were collected without space. Based on the voxel, the dense zone regions of the upper, middle, and lower parts of the substantia nigra were delineated to measure the signal changes of the corresponding regions to achieve a quantitative analysis of iron deposition in the dense zone of the substantia nigra. In the image processing, six cases in the PD group had partial plane motion, resulting in angle deviation, which could not be corrected for fusion measurement. Thus, 36/42 patients in the PD group were included.

After the data of all sub-regions are counted, the amplitude, phase value and R2^*^ value of the three sub-regions of the substation nigra compact zone on both sides are added respectively, and then the average value is calculated as the signal representation of the overall substation nigra compact zone compact zone on both sides, and the energy analysis is made for each average value parameter to help understand the impact of iron deposition on the overall substation nigra compact zone.

### 2.4. Statistical analysis

An independent sample *t*-test or chi-square test was used for comparisons between the two groups. Pearson's correlation analysis was used to evaluate the correlation between each sequence of imaging indicators and clinical indicators in PD. All statistical analyses were performed using SPSS Statistics software (version 22.0). Statistical significance was set at *P* < 0.05.

## 3. Results

### 3.1. Subject characteristics

Among the MR images of 42 patients, images could not be fused for quantitative analysis due to poor image quality in 6 patients. Therefore, 36 patients (16 males and 20 females) in the PD group were included in the final analysis. The average patient age was 70.28 ± 13.14 years, the average disease duration was 6.48 ± 6.09 years, and the average H–Y grade was 2.76 ± 1.07. Meanwhile, the control group (*n* = 36) included 17 males and 19 females, and the average patient age was 63.83 ± 14.41 years. There was no significant between-group difference in age (*P* = 0.051).

### 3.2. Multi-parameter MRI fusion image analysis

#### 3.2.1. Comparison of iron deposition in the upper, middle, and lower parts of the substantia nigra compacta zone and average values between the two groups based on fusion images

There were significant between-group differences in the amplitude (amplitude: *t* = −2.138, *P* < 0.05), phase value (*t* = −2.802, *P* < 0.05), and R2^*^ value (*t* = 2.424, *P* < 0.05) in the upper part of the right substantia nigra compact zone. Meanwhile, only the amplitude (*t* = −2.711, *P* < 0.05) but not the phase value (*t* = −1.680, *P* > 0.05) and R2^*^ value (*t* = 1.992, *P* = 0.05) in the middle of the right substantia nigra dense zone was significantly different between the two groups. Similarly, there were significant between-group differences in the amplitude (*t* = −2.019, *P* < 0.05) but not in the phase value (*t* = −0.808, *P* > 0.05) and R2^*^ value (*t* = 0.642, *P* > 0.05) in the lower part of the right substantia nigra dense zone.

In contrast, in the upper part of the left substantia nigra compacta, the phase value (*t* = −2.242, *P* < 0.05) and R2^*^ value (*t* = 2.018, *P* < 0.05), but not the amplitude (*t* = −1.248, *P* > 0.05), were significantly different between the two groups. For the middle of the left substantia nigra dense zone, there was a significant between-group difference in amplitude (*t* = −2.039, *P* < 0.05) but not in phase value (*t* = −0.760, *P* > 0.05) and R2^*^ value (*t* = 1.336, *P* > 0.05). For the lower part of the left substantia nigra compacta zone, there were no significant between-group differences in amplitude (*t* = −1.670, *P* > 0.05), phase value (*t* = −1.266, *P* > 0.05) and R2^*^ value (*t* = 0.472, *P* > 0.05).

**Figure 4:**
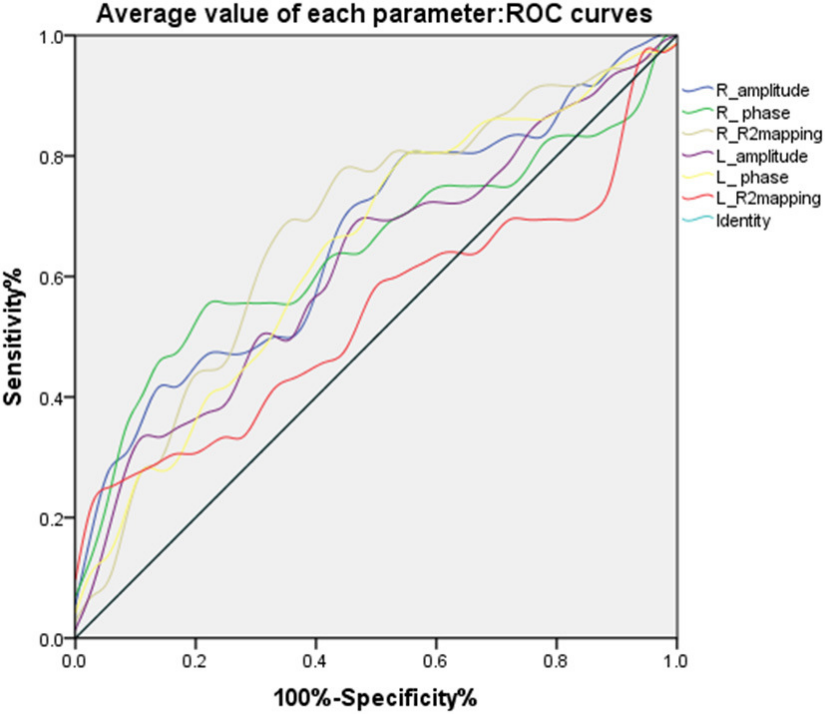
ROC curves of the average values of different parameters in bilateral substantia nigra compact zone.

Meanwhile, the average values in the right substantia nigra compacta (amplitude: *t* = −2.747, *P* < 0.05, phase value: *t* = −2.338, *P* < 0.05, R2^*^ value: *t* = 2.334, *P* < 0.05) was significantly different among the three sub-regions. For the overall average values in the left substantia nigra compact zone, only phase was significantly different between the two groups (amplitude: *t* = −1.892, *P* > 0.05; phase value: *t* = −2.197, *P* < 0.05; and R2^*^ value: *t* = 1.200, *P* > 0.05) (Table 2). In addition, the ROC curve was used to analyze these average values, and it was found that the R2^*^ value on the right side (AUC = 0.678) has a high diagnostic efficiency for the detection of iron deposition in the substation nigra compact zone of PD, and the calculation of threshold value of each index (Figure 4 and Table 3).

**Table 2:** Based on the comparison between the upper, middle and lower three sub regions and the average iron deposition groups of SNc.

	**Group (mean** ±**standard deviation)**		***t***	***P***
	**PD (*****n*** = **36)**	**Healthy control group (*****n*** = **36)**		
Amplitude of upper R-SNc	90.17 ± 12.04	95.76 ± 10.07	−2.138	0.036
Phase value of upper R-SNc	−0.0463 ± 0.0580	−0.0135 ± 0.0396	−2.802	0.007
R2^*^ value of upper R-SNc	31.73 ± 3.89	29.63 ± 3.46	2.424	0.018
Amplitude of middle R-SNc	85.73 ± 11.29	92.13 ± 8.56	−2.711	0.008
Phase value of middle R-SNc	−0.0326 ± 0.0772	−0.0081 ± 0.0407	−1.680	0.097
R2^*^ value of middle R-SNc	31.40 ± 4.25	29.65 ± 3.15	1.992	0.050
Amplitude of lower R-SNc	96.44 ± 11.61	101.65 ± 10.26	−2.019	0.047
Phase value of lower R-SNc	−0.0353 ± 0.1007	−0.0190 ± 0.0682	−0.808	0.422
R2^*^ value of lower R-SNc	27.61 ± 4.79	26.92 ± 4.21	0.642	0.523
Amplitude of upper L-SNc	92.24 ± 9.19	94.78 ± 8.05	−1.248	0.216
Phase value of upper L-SNc	−0.0399 ± 0.0459	−0.0186 ± 0.0337	−2.242	0.028
R2^*^ value of upper L-SNc	30.39 ± 4.19	28.67 ± 2.93	2.018	0.047
Amplitude of middle L-SNc	86.96 ± 10.04	91.55 ± 9.06	−2.039	0.045
Phase value of middle L-SNc	−0.0170 ± 0.0576	−0.0079 ± 0.0420	−0.760	0.450
R2^*^ value of middle L-SNc	29.86 ± 4.01	28.78 ± 2.74	1.336	0.186
Amplitude of lower L-SNc	94.54 ± 12.53	99.12 ± 10.67	−1.670	0.099
Phase value of lower L-SNc	−0.0677 ± 0.0804	−0.0463 ± 0.0613	−1.266	0.210
R2^*^ value of lower L-SNc	26.22 ± 4.65	26.67 ± 3.43	−0.472	0.639
Average amplitude of R-SNc	90.50 ± 10.24	96.51 ± 8.23	−2.747	0.008
Average phase value of R-SNc	−0.0381 ± 0.0548	−0.0135 ± 0.0311	−2.338	0.022
Average R2^*^ value of R-SNc	30.25 ± 2.87	28.73 ± 2.63	2.334	0.022
Average amplitude of L-SNc	91.24 ± 9.33	95.15 ± 8.14	−1.892	0.063
Average phase value of L-SNc	−0.0415 ± 0.0358	−0.0243 ± 0.0305	−2.197	0.031
Average R2^*^ value of L-SNc	28.82 ± 3.40	28.03 ± 1.93	1.200	0.234

Independent-sample t-test; R, right; L, left.

**Table 3:** Analysis of ROC curves for average value of each parameter.

**Parameter average**	**Sensitivity**	**Specificity**	**AUC**	***P***	**Threshold**	**95%CI**
R_amplitude	41.7%	88.9%	0.664	0.011	87.23333	0.543~0.771
R_phase	55.6	80.6	0.645	0.032	−0.0339	0.523~0.754
R_R2^*^mapping	77.8	58.33	0.678	0.006	28.37	0.557~0.783
L_amplitude	33.3	91.7	0.621	0.070	85.8667	0.499~0.733
L_phase	77.8	50.0	0.644	0.028	−0.0187	0.523~0.754
L_R2^*^mapping	25.0	97.2	0.538	0.592	30.8733	0.416~0.656

R, right; L, left.

#### 3.2.2. Correlation analysis of PD patients

**Figure 5:**
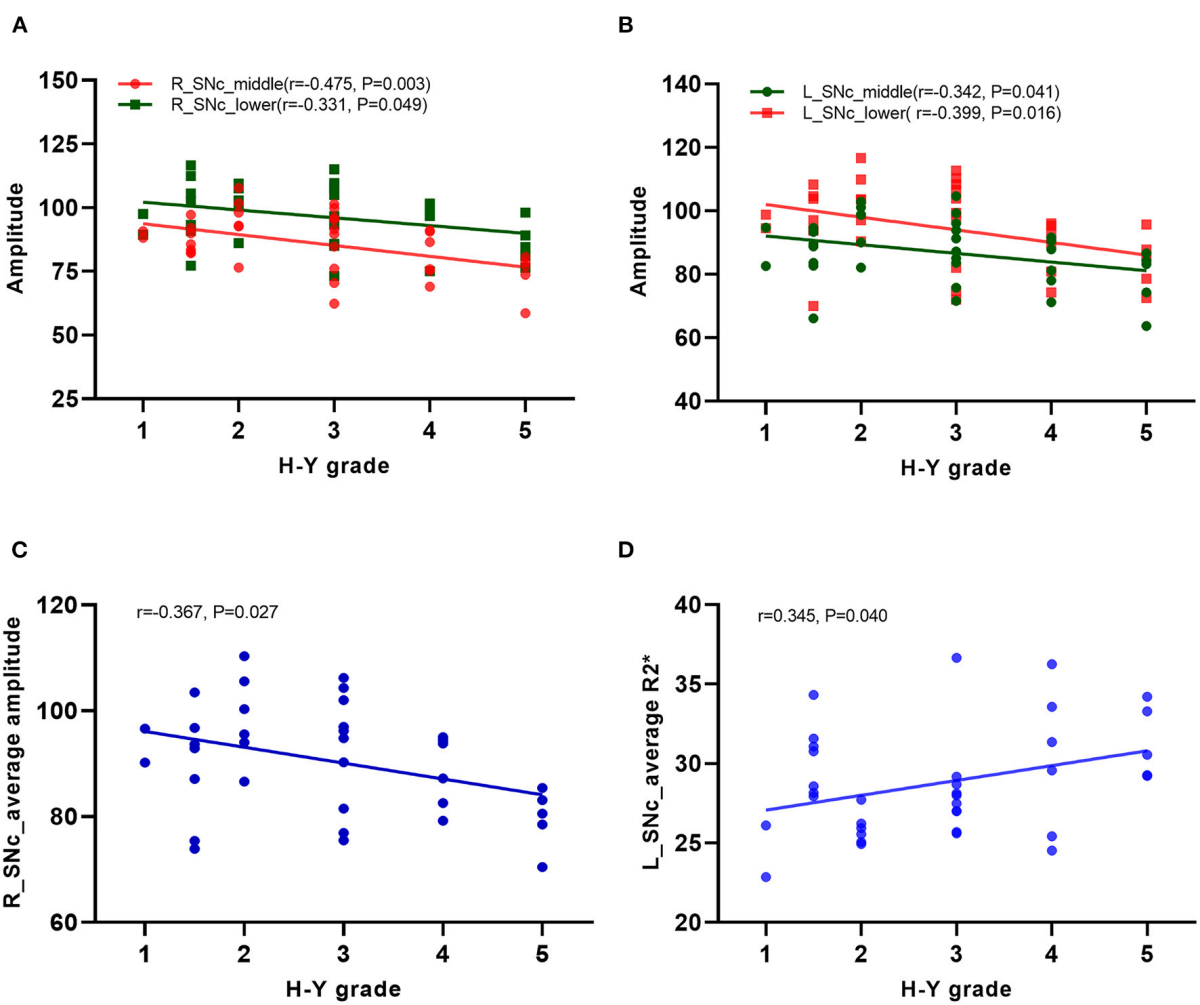
Correlation analysis in PD patients. **(A)** Correlation analysis between the amplitudes of the middle and lower parts of the right side of the substation nigra compact zone and H–Y scale; **(B)** Correlation analysis between the amplitudes of the middle and lower parts of the left side of the substation nigra compact zone and H–Y scale; **(C)** Correlation analysis between the average amplitude on the right side of the substation nigra compact zone and H–Y scale; **(D)** Correlation analysis between the the average R2* value on the left side of the substation nigra compact zone and H–Y scale.

In the PD group, the correlation of the amplitude, phase, and R2^*^ values of each sub-region in the substantia nigra compact zone with disease onset time and H–Y grade was analyzed using Pearson correlation analysis. The results showed that the amplitudes of the middle and lower parts of the right side of the substantia nigra compacta zone were negatively correlated with the H–Y grade (middle: *r* = −0.475, *P* = 0.003; lower: *r* = −0.331, *P* = 0.049, Figure 5A), while those in the left side of the substantia nigra compacta zone were negatively correlated with the H–Y grade (middle: *r* = −0.342, *P* = 0.041, lower: *r* = −0.399, *P* = 0.016, Figure 5B). In addition, Pearson correlation analysis was performed for the correlation between the average value of amplitude, phase value, and R2^*^ value of the three sub-regions of the substantia nigra dense with disease onset time and H–Y grade. The results showed that the average amplitude on the right side of the substantia nigra compacta zone was negatively correlated with the H–Y grade (*r* = −0.367, *P* = 0.027, Figure 5C), while the average R2^*^ value on the left side of the substantia nigra compacta zone was positively correlated with the H–Y grade (*r* = 0.345, *P* = 0.040, Figure 5D). In addition, the onset time of PD was positively correlated with the H–Y scale grade (*r* = 0.396, *P* = 0.017).

## 4. Discussion

Histologically, neurons in the dense zone of the substantia nigra have rich melanin and poor iron content. In contrast, the cells in the reticular zone of the substantia nigra are rich in iron but not in melanin. Previous physiological, pathological, and anatomical studies have confirmed that the neuronal degeneration in the substantia nigra compacta in patients with PD is closely related to an abnormal increase in iron content. Therefore, quantitative analysis of iron deposition in the substantia nigra compact zone is particularly important for understanding the pathophysiological changes of neuronal degeneration in PD patients (12–15).

There are currently many quantitative analysis studies for iron deposition in the substantia nigra using magnetic resonance methods, such as SWI, QSM, and T2^*^mapping (14–16). However, these single sequences cannot distinguish the dense zone of the substantia nigra from the reticular zone during measurement, especially when the iron deposition in the dense zone of patients with PD increases abnormally. In addition, these sequences are sensitive to iron deposition but cannot distinguish their boundaries well. Therefore, the dense and reticular zones of the substantia nigra are mostly used to analyze the iron deposition content to judge the degree of neuronal degeneration (17, 18).

To better understand iron deposition and distribution in the substantia nigra compact zone, as well as their correlation with the course and severity of the disease, this study used NM-MRI to better display the distribution of melanin neurons in the substantia nigra, so as to better locate the substantia nigra compact zone. NM-MRI of the same case was fused with SWI and T2^*^mapping sequences, which can accurately delineate iron deposition by SWI and T2^*^mapping sequences in the distribution area of melanin neurons, to achieve a quantitative analysis of iron deposition in the dense zone of the substantia nigra (19). To better understand the distribution of iron deposition in the substantia nigra compact zone, the area of the substantia nigra compact zone was equally divided into three regions, and iron deposition in each region was analyzed.

The results showed that iron deposition in the upper part of the right substantia nigra compact zone was significantly higher in the PD group than in the control group, and the amplitude, phase value, and R2^*^ values were also significantly different. In contrast, in the middle and lower parts of the right substantia nigra compact zone, only the amplitude was significantly different between the two groups, and the phase value and R2^*^ value was not. This may be because the amplitude is more sensitive to the iron deposition measurement than is the phase value and R2^*^ value in the middle and lower parts of the substantia nigra compact zone. Another reason is because the difference between the phase value and R2^*^ value cannot be observed when the iron deposition content in the substantia nigra compact zone is smaller than that in the reticular zone.

Compared with the normal control group, the PD group showed significantly higher iron deposition in the upper part of the left substantia nigra compact zone, and the phase and R2^*^ values were also significantly different. Meanwhile, for the middle of the left substantia nigra compact zone, only the amplitude value, but not the phase value and R2^*^ value, showed significant differences. The lower part of the left substantia nigra compact zone did not show significant changes in iron deposition, and the amplitude, phase, and R2^*^ values were not significantly different between the two groups. Collectively, these results indicated more significant neuronal degeneration in the substantia nigra of PD patients or a top-down degeneration process of PD. The research of Du Guangwei et al. is based on the analysis of the whole substantia nigra dense zone and reticular zone. It is mentioned that R2^*^ and quantitative susceptibility map are higher in the substantia nigra dense zone and reticular zone of all PD patients (20). Arribarat et al. (21) divided the substation nigra into anterior and posterior parts for analysis, showing that the relaxometry T2^*^ values were greater for PD patients than HCs in the anterior SN, but not in relation with the iron deposition in the posterior SN with the relaxometry T2^*^. This also shows that the results of comparison between various parameters and the sensitivity to iron deposition are different in the overall analysis of substation nigra compact and the analysis of sub regions. This study will compare and analyze different parameters and indicators in different sub regions, observe the parameters in each sub region of substation nigra compact zone. Through the study of iron deposition in each sub-region, the influence of iron deposition in the substantia nigra compact zone on melanin neurons and whether the change of iron deposition in each sub-region is related to the occurrence and development of disease were understood. The sensitivity of each parameter to its internal iron deposition was compared and analyzed, providing a reference for the subsequent research in the sub-region.

To understand the effect of iron deposition on neuronal degeneration in the entire substantia nigra compact zone, the mean of the measured values in Sanya were used in the analysis. The results showed that the mean values of amplitude, phase, and R2^*^ of the right substantia nigra compact zone were significantly different between the PD and healthy control groups. This indicates that the overall iron deposition in the substantia nigra compact zone was more significant in the PD group than in the healthy control group, consistent with previous findings. For values in the left substantia nigra compact zone, the phase value, but not the amplitude and R2^*^ values, were significantly different between the two groups. The overall analysis indicates that the phase value is more sensitive to iron deposition than is the amplitude and R2^*^ values. Du Guangwei et al. also mentioned in the study that during the 18 months follow-up, the substantia nigra pars compacta R2^*^ showed a faster increase in PD compared with controls. Through ROC curve analysis, we found that the average value of R2^*^ in the right substantia nigra compact zone has higher diagnostic energy efficiency, in line with previous results (2, 20, 22).

The study also analyzed the correlation of the measured values of each sub-region and the whole dense zone of the substantia nigra with the course and degree (H–Y grade) of the disease. The results showed that the amplitude of the middle and lower parts of the bilateral substantia nigra dense zone was negatively correlated with the H–Y stage, while the value measured at the upper part was not correlated with the disease course and degree. This indicates that the more iron deposition in the middle and lower parts of the substantia nigra dense zone, the more severe is PD. Moreover, the change in iron deposition in the middle and lower parts of the substantia nigra dense zone may highlight the severity of PD. Correlation analysis of the overall average value of the substantia nigra dense zone with the disease course and degree (H–Y grade) showed that the average amplitude value in the right and the average R2^*^ value in the left of the substantia nigra dense zone were correlated with the H–Y grade. This supported that an abnormal increase in iron deposition in the substantia nigra dense zone was correlated with the severity of PD (23). Different measurement methods obtained the same results, consistent with previous results (24, 25).

The study findings show that iron deposition in the upper part of the dense zone of the substantia nigra is more obvious in the PD group than in the healthy control group, but the severity of PD could not be evaluated. The results of quantitative analysis of iron deposition in the middle and lower parts of the right substantia nigra compact zone and in the middle part of the left substantia nigra compact zone both showed a difference in iron deposition according to PD severity. This indicated that the analysis of iron deposition in sub-regions is helpful for early PD diagnosis and the evaluation of disease progression. In particular, the amplitude value is sensitive to quantitative iron deposition. The amplitude, phase, and R2^*^ values of the three sub-regions showed significant differences in iron deposition in PD patients. The right amplitude and left R2^*^ value was correlated with disease severity in PD patients. This indicated that iron deposition in the overall or sub-regions of the substantia nigra compact zone is related to neuronal degeneration in this zone, and thus, its analysis clinically significant for the evaluation of disease in PD patients (6). The analysis and comparison of different measurement methods of iron deposition highlight the clinical value of different parameters, which can provide a basis for more accurate imaging biological indicators for clinical PD diagnosis (26, 27).

This study had some limitations. First, patients were required to be highly cooperative during the examination process. Only when a complete sequence is acquired at one time and the image meets the diagnostic requirements can the subsequent image fusion process be guaranteed. The resolution was relatively high, and the acquisition time was relatively long. Second, in the fusion process, the required sequence images are fused in pairs, not all sequence parameters are fused together. Because the distribution of high signals in the substantia nigra dense zone varies greatly among individuals, the images are manually delineated by the high signal areas on the NM-MRI image, which cannot be analyzed based on voxels or atlases, and it is inevitable to cause certain deviation to the results. However, we also involved two physicians to jointly confirm and delineate the regions of interest to reduce the artificial influence on the results. Third, due to individual differences in the size of the substantia nigra dense zone, layer selection was measured at the defined sub-region level. Some parts of the substantia nigra dense zone with larger or smaller volumes that exceeded the sub-region were measured twice, and the average value of the lower sub-region was included in the analysis, which may have a certain impact on the results. However, the two physicians discussed the findings during data processing to reach an agreement, minimizing this impact.

In summary, this study improved the method of magnetic resonance multiparameter display in the substantia nigra dense zone area and iron deposition measurement and analysis to evaluate iron distribution and deposition in the substantia nigra dense zone. This can help PD diagnosis and the evaluation of disease severity. Further, exploratory research was conducted to identify additional biological imaging indicators for clinical PD diagnosis, with the value of each indicator in the clinical diagnosis and evaluation of PD described.

## Data availability statement

The original contributions presented in the study are included in the article/supplementary material, further inquiries can be directed to the corresponding authors.

## Ethics statement

The studies involving human participants were reviewed and approved by the Ethics Committee of Guangzhou Xinhai Hospital. The patients/participants provided their written informed consent to participate in this study. Written informed consent was obtained from the individual (s) for the publication of any potentially identifiable images or data included in this article.

## Author contributions

All authors listed have made a substantial, direct, and intellectual contribution to the work and approved it for publication.
